# The evolution of nursing in Australian general practice: a comparative analysis of workforce surveys ten years on

**DOI:** 10.1186/1471-2296-15-52

**Published:** 2014-03-25

**Authors:** Elizabeth J Halcomb, Yenna Salamonson, Patricia M Davidson, Rajneesh Kaur, Samantha AM Young

**Affiliations:** 1 School of Nursing & Midwifery, University of Wollongong, Sydney, Australia; 2 School of Nursing & Midwifery, University of Western Sydney, Sydney, Australia; 3 Johns Hopkins University School of Nursing, Baltimore, USA; 4 University of New South Wales, Sydney, Australia; 5 University of Newcastle, Sydney, Australia

**Keywords:** Practice nurse, Nursing workforce, Survey, Office nurse, General practice, Primary care, Australia

## Abstract

**Background:**

Nursing in Australian general practice has grown rapidly over the last decade in response to government initiatives to strengthen primary care. There are limited data about how this expansion has impacted on the nursing role, scope of practice and workforce characteristics. This study aimed to describe the current demographic and employment characteristics of Australian nurses working in general practice and explore trends in their role over time.

**Methods:**

In the nascence of the expansion of the role of nurses in Australian general practice (2003–2004) a national survey was undertaken to describe nurse demographics, clinical roles and competencies. This survey was repeated in 2009–2010 and comparative analysis of the datasets undertaken to explore workforce changes over time.

**Results:**

Two hundred eighty four nurses employed in general practice completed the first survey (2003/04) and 235 completed the second survey (2009/10). Significantly more participants in Study 2 were undertaking follow-up of pathology results, physical assessment and disease specific health education. There was also a statistically significant increase in the participants who felt that further education/training would augment their confidence in all clinical tasks (p < 0.001). Whilst the impact of legal implications as a barrier to the nurses’ role in general practice decreased between the two time points, more participants perceived lack of space, job descriptions, confidence to negotiate with general practitioners and personal desire to enhance their role as barriers. Access to education and training as a facilitator to nursing role expansion increased between the two studies. The level of optimism of participants for the future of the nurses’ role in general practice was slightly decreased over time.

**Conclusions:**

This study has identified that some of the structural barriers to nursing in Australian general practice have been addressed over time. However, it also identifies continuing barriers that impact practice nurse role development. Understanding and addressing these issues is vital to optimise the effectiveness of the primary care nursing workforce.

## Background

A practice nurse (PN), is a registered or an enrolled nurse who provides nursing services within a general practice setting. Practice nurses can be either registered nurses (RN), who are baccalaureate prepared, or enrolled nurses (EN), who have undertaken diploma level training [1,2]. These differences in educational preparation impact on the regulated scope of the nurses clinical practice. The general practice nurse is not as well recognised as an independent nursing specialty in Australia [3], as it is in the United Kingdom (UK) and New Zealand (NZ) [4,5]. The general practice nurse in the UK has evolved from a task-oriented position to a key player within an integrated, multidisciplinary primary care team [6]. A major distinction between the current state of nursing in general practice within the UK and NZ and the current Australian role is the presence of career frameworks, comprised of salary structures and levels of nursing practice which articulate roles based on the nurses experience, education and scope of practice [7]. In Australia, there remains no defined career pathway [7] and PN roles have been demonstrated to often be linked to funding schemes that provide reimbursement for specific activities [8,9].

The nursing role in Australian general practice has undergone significant expansion over the past decade. Changes in health policy, funding models and nurse education are transforming the landscape of Australian primary care [10,11]. Policy makers are seeking to build sustainable primary care services to reduce the burden of chronic and complex disease. Financial incentives are being offered to provide evidence based care for specific disease groups, many of which are nurse-led. Nurse education providers are increasingly seeking to prepare graduates to work in primary care and to provide postgraduate courses with a primary care focus [12]. This transformative agenda is being driven by the increasing burden of non-communicable diseases, a need for improved coordinated management of chronic and complex conditions and the increasing evidence for the value of preventative care [13]. Monitoring and responding to both push and pull factors in the health workforce is important in ensuring a dynamic and responsive primary care workforce.

In 2003, 40% of Australian general practices employed a nurse and it was estimated that there was 2349 nurses employed in Australian general practice [14]. In response to policy change this number grew rapidly, and in 2008, it was estimated that there were approximately one nurse per 2.3 general practitioners [3,14], a ratio similar to that of NZ [3]. By 2009, 56.9% of Australian general practices were reported to employ one or more of the 8914 estimated nurses now working in Australian general practice [15]. Such rapid workforce growth has significant implications for both nurses, the workforce as a health care team and the system within which they practice.

Several investigations have sought to examine the Australian PN workforce at various points in its evolution. In the early period, Patterson [16] undertook a case study of the role of nurses employed within a single region of general practices. This study described differences in perception of the nature of the nursing role in general practice between general practitioners and nurses. Through this work, Patterson [16] identified a lack of understanding of the boundaries of the nurses scope of practice. Several years later the Royal Australian College of General Practitioners and the Royal College of Nursing, Australia sought to investigate the roles of nurses working in general practice across Australia [17,18]. Whilst this study identified a diversity of roles within Australian general PNs, they found four common elements, namely; clinical care; clinical organization; practice administration and integration of the practice with external organizations [17,18]. This work also identified a number of factors that impacted on the current and future practice nurse role, including lack of education pathways, clarification of nurse roles, systems issues, legal, funding and workforce issues [18]. Whilst these factors were identified, the nature of the impact which they had on the nurses’ role was unclear.

More recently, Joyce & Piterman [3,19,20] have examined the nurses role in Australian general practice. This work involved a cross-sectional national survey which explored nurse demographics, work environments and duties [19] and nurse-patient encounters in Australian general practice [3,20]. This work identified a gap in knowledge around nurses’ roles in patient care and a need for better monitoring of the practice nurse workforce [19]. This literature provides important insights into the various stages of the evolution of practice nursing in Australia. However, in order to truly appreciate how the workforce has evolved it is important to trace the trends in workforce characteristics, roles and the work environment over time. In this paper, we compare and contrast the findings of two Australian investigations of the practice nurse workforce to examine the evolution in demographic, work roles and employment characteristics of practice nurses over the past ten years.

## Methods

### Design

A national cross-sectional survey of nurses employed in general practice was conducted during both 2003–04 and 2009–10 using a structured survey tool as a part of two larger mixed methods investigations of the clinical roles of these nurses [8,21]. This paper provides a comparative analysis of the data from these two surveys to explore the trends in workforce development over time.

### Sample/participants

As there is no central or local register that identifies practice nurses, both surveys used a multifaceted approach to participant recruitment. Nurses working in general practice were recruited to complete the survey from delegates attending the Australia General Practice Nursing Conference, via advertisements disseminated through the Divisions of General Practice, Australian Practice Nurses Association and State/Territory industrial nursing organisations or via direct email from Divisional practice nurse program staff. Potential participants who contacted the research team were either sent a copy of the information sheet and survey form directly from the research team or via Divisions of General Practice or Australian Practice Nurses Association. Links to the survey form and advertisements about the research were also placed on relevant professional websites and in relevant professional publications. Email reminders were sent to all potential participants who provided contact details to the research team and to the Divisional Staff who facilitated survey distribution. Despite the limitations of such convenience sampling, the lack of employment data precluded the use of more representative sampling techniques.

The first survey involved 284 nurses employed in general practice across six Australian states. These data have been previously reported [8,21]. The second survey involved 235 nurses employed in general practice across six Australian states.

### Survey tool

For the first study, a survey tool was developed following a review of the literature and key informant consultation. This tool was pilot tested with 14 respondents before widespread distribution. This method has been previously reported [8]. The survey was comprised of three sections; (i) demographic, employment and workplace characteristics, (ii) barriers and facilitators to role expansion, and (iii) the clinical role. The third section provided a list of clinical tasks and asked participants to identify tasks that they currently undertook in their practice, tasks that they felt were appropriate for a nurse in general practice and tasks for which they felt they required additional education/training. These tasks were selected to represent the kinds of activities that a nurse might contribute to in terms of their role in the assessment, ongoing management and self-management support of individuals with chronic disease.

The second survey comprised of items repeated from the first survey and some additional items related specifically to chronic disease management. This paper reports the data from the items which were collected in both surveys.

### Ethical considerations

The Human Research Ethics Committee of the University of Western Sydney granted approval for the conduct of both surveys (Approval No. HEC 03/166 & H6774) before the commencement of data collection. Return of the completed survey form was considered indication of the participants consent to participate.

### Data analysis

All data analyses were executed using the SPSS Version 21.0 software. Descriptive statistics were summarised using frequencies and percentages for categorical variables, frequencies, mean, standard deviations and ranges for continuous variables. Inferential statistical analyses were also undertaken. Distributions of continuous variables were first checked for normality using Smirnov-Kolmogorov test. The Mann–Whitney U test was used to assess for group differences of continuous variables that were not normally distributed (independent t-test for normally distributed continuous variables), and Pearson chi-square test for group differences in categorical variables. The p<0.05 value was set as the cut-off for statistical significance.

### Validity and reliability

The content validity of the tool was established in the first survey by a panel of clinical nurses and research experts. A further panel of experts reviewed the second survey instrument prior to survey administration.

## Results

### Participant demographics

Table 1 summarises the sociodemographic and practice related variables of the two data collections. Whilst similar recruitment methods were employed in both surveys, a slightly smaller sample size was achieved in study 2 (284 versus 235) despite a growth in the overall population of nurses working in general practice. This should be considered in the interpretation of these data. Participants in study 2 were slightly, but not significantly, older than those in study 1 (mean age 47.52 yrs versus 45.83 yrs; *p*=0.022). Whilst only one male nurse responded to the first study, study 2 included 8 male respondents (*p*=0.007). The number of enrolled nurse participants more than doubled from study 1 to study 2 (6.3% versus 14.0%). Additionally, participants in Study 2 were more likely to hold an advanced certificate or tertiary qualification (54.3% versus 35.5; *p*<0.001).

**Table 1:** Socio-demographic characteristics of participants in two studies

**Characteristic**	**Study 1 (2003/04) n = 284**	**Study 2 (2009/10) n = 235**	**p**
Age (mean *SD*), years	45.83 (7.30)	47.52 (9.26)	0.022^a^
Sex (Female)%	99.6	96.5	0.007^c^
Hours per week as PN (mean *SD*) hours	26.22 (9.80)	26.84 (14.39)	0.89^b^
Nursing Classification %			
Non-nursing	2.1	2.1	0.02^c^
Enrolled nurse	6.3	14	
Registered nurse	85.6	77	
Clinical nurse specialist/Clinical nurse consultant/Nurse manager	6.0	6.8	
Nursing qualification %			
Hospital trained	64.5	45.7	<0.001^c^
Advanced certificate or tertiary education	35.5	54.3	
Years of practice as a qualified nurse (mean *SD*)	20.58 (8.28)	21.58 (9.97)	0.219^a^
Duration worked as PN (mean *SD*) years	7.51 (6.86)	6.40 (6.38)	0.068^b^
Locality of practice %			
Inner city/urban	38.1	44.2	0.096^c^
Rural/Regional	40.6	33.8	
Rural/Remote	19.8	22.1	
Postcode by practice %			
NSW	44.4	36.8	<0.001^c^
Victoria	20.1	7.3	
Queensland	17.2	15.9	
South Australia	5.4	22.3	
Western Australia	9.7	15.5	
Tasmania	3.2	2.3	
Own room/treatment area %	94.7	91.2	0.121^c^
Current policy/procedure manual (Yes) %	73.0	77.6	0.241^c^

Note: ^a^Independent samples *t* test/^b^Mann–Whitney *U* test (non-normal distribution of scores)/^c^ Pearson *χ*^2^ test.

Both datasets included responses from the 6 Australian States, however, there were more respondents from South Australia (22.3% versus 5.4%) and significantly less from NSW (36.8% versus 44.4%) and Victoria (7.3% versus 20.1%) in study 2 compared to study 1 (*p*<0.001). Despite this geographical variation there was no significant difference in the locality of practice between the two datasets (p=0.096), with a mix of rural, inner city and remote practice nurses participating in both surveys.

Overall no significant differences were found between hours worked per week as PN, years of practice as qualified nurse and years worked as a practice nurse in both studies. This finding is interesting given the passing of time between the studies. If nurses were retained in the workforce it would be expected that the years worked as a practice nurse would increase between the two studies. This finding adds further weight to the anecdotal evidence of significant turnover within the practice nurse workforce and issues of retention of nurses. These data also demonstrate that the predominance of part-time workers within the workforce that has maintained steady across the two time periods.

### Nurse roles in general practice

Participants were provided a list of clinical activities and a matrix to identify which activities they felt were appropriate tasks for nurses within general practice, which tasks they currently undertook within their practice and which tasks they felt that they needed additional education or training in order to be confident. There was a small but non-significant rise in the number of participants in the two surveys who felt that the clinical activities identified were appropriate tasks for practice nurses (Table 2). Data from the initial study demonstrated that roles of nurses employed in general practice focussed on core clinical skills that attracted remuneration for the Practice, such as wound dressings, immunisation. In contrast, Table 2 demonstrates the broader services now being delivered by practice nurses. A statistically significant increase was observed in the number of participating nurses undertaking follow-up of pathology results (p=0.001), physical assessment (p<0.001), and providing disease specific health education (p<0.001).

**Table 2:** Clinical activities undertaken

**Clinical activity**	**Do you think this is an appropriate activity for a practice nurse?**					**Do you undertake this activity in your clinical practice?**				
	**Study 1**		**Study 2**		**p**	**Study 1**		**Study 2**		***p***
	**n**	**%**	**n**	**%**		**n**	**%**	**n**	**%**	
Vital signs measurement	265	*95.7*%	219	*93.2*%	0.219	256	*91.8*%	208	*88.5*%	0.216
Follow up of pathology results	172	*62.1*%	174	*74.0*%	0.004	132	*47.3*%	147	*62.6*%	0.001*
ECG testing	257	*92.8*%	217	*92.3*%	0.850	241	*86.4*%	188	*80.0*%	0.052
Physical Assessment	213	*76.9*%	184	*78.3*%	0.705	127	*45.5*%	148	*63.0*%	<0.001*
Counselling for mental health issues	162	*58.5*%	96	*40.9*%	<0.001*	87	*31.2*%	45	*19.1*%	0.002*
Disease-specific health education	210	*75.8*%	197	*83.8*%	0.025	120	*43.0*%	158	*67.2*%	<0.001*
Assessment of social support	205	*74.0*%	181	*77.0*%	0.430	125	*44.8*%	126	*53.6*%	0.046
Assessment of medication regimes	133	*48.0*%	116	*49.4*%	0.761	74	*26.5*%	67	*28.5*%	0.615
Case-management/Co-ordination	152	*54.9*%	153	*65.1*%	0.019	79	*28.3*%	90	*38.3*%	0.016

*statistically significant.

**Figure 1:**
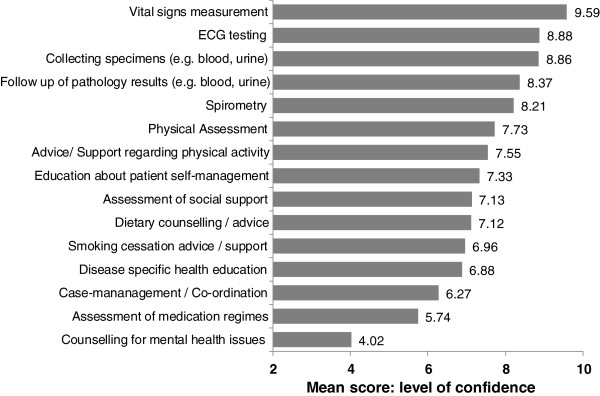
Mean confidence with clinical activities.

The only clinical activity that was reportedly significantly less frequently undertaken was counselling for mental health issues (p=0.002). Significantly fewer participants in study 2 felt that this was an appropriate task for nurses within general practice (p<0.001). This finding may be related to the recent introduction of specialist mental health nursing services to Australian primary care. Additionally, as can be seen from Figure 1, this was the clinical activity which participants rated themselves as being least confident. However, level of confidence on a 10-point Likert scale did not completely explain whether or not participants undertook an activity. Whilst over half of participants (n=152; 54.9% and n=153; 65.1%) reported feeling that undertaking case management was within their role, the mean level of confidence in undertaking this task was only 6.27.

When asked whether further education/training would increase their confidence in undertaking each activity, a statistically significant increase was noted for each clinical activity between the two datasets (Table 3). These data confirmed the areas in which nurses within general practice had identified as those in which they were last confident to practice.

**Table 3:** Need for further education/training

**Clinical activity**	**Would further education/training increase your confidence in undertaking this activity?**				
	**Study 1**		**Study 2**		***p***
	**n**	**%**	**n**	**%**	
Vital signs measurement	10	3.6%	47	32.9%	<0.001*
Follow up of pathology results	68	24.4%	91	38.7%	<0.001*
ECG testing	37	13.4%	73	31.1%	<0.001*
Physical Assessment	83	30.1%	117	49.8%	<0.001*
Counselling for mental health issues	122	43.7%	147	62.6%	<0.001*
Disease-specific health education	125	45.3%	142	60.4%	0.001*
Assessment of social support	83	30.1%	119	50.6%	<0.001*
Assessment of medication regimes	104	37.7%	141	60.0%	<0.001*
Case-management/Co-ordination	96	34.8%	127	54.0%	<0.001*

*statistically significant.

### Barriers to role development

**Figure 2:**
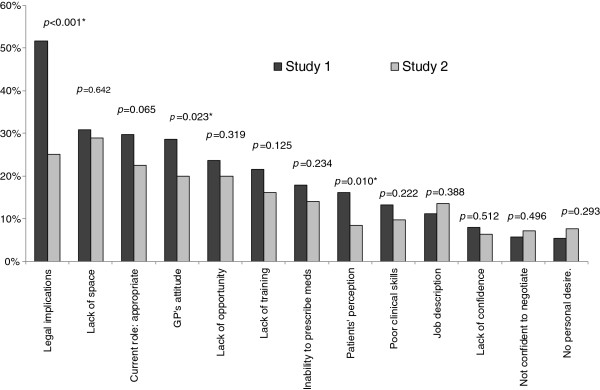
Barriers to the expansion of practice nurses’ role.

As can be seen from Figure 2, the barriers to the expansion of the nurses’ role in general practice have changed in many ways between the two studies. Three barriers, in particular, were seen as much less of a barrier to role expansion in study 2 than they had been in study 1. Firstly, whilst slightly more than half the participants in study 1 (51.6%) reported legal implications as a barrier, only a quarter of participants in study 2 (25.1%; *p*<0.001) felt that this still negatively impacted role development for nurses within general practice. Secondly, patient’s perceptions of nurses’ role expansion improved significantly. Compared to study 1 (16.1%) half the number of nurses’ in study 2 considered patient’s perceptions of their role as a barrier to their role development (8.5%; *p*=0.01).

Additionally, the impact of general practitioners (GPs) attitude on the role of nurses within general practice development has changed significantly. Whilst 28.7% of participants in study 1 saw this as a barrier, only 20% reported this as an impediment in study 2 (28.7%; *p=*0.02). However, a number of barriers related to the GP were consistent across the two surveys. These included; GPs not understanding the nurses’ scope of practice, lack of teamwork between GPs and nurses’, unwillingness of some GPs to delegate tasks to the nurse, and variation in practice between GPs.

Conversely, three barriers were identified by more respondents in study 1 than in study 2, namely lack of job description, low confidence to negotiate with general practitioners, and a lack of the nurses’ personal desire to enhance their role. None of these differences were statistically significant (Figure 2).

### Facilitators of role development

**Figure 3:**
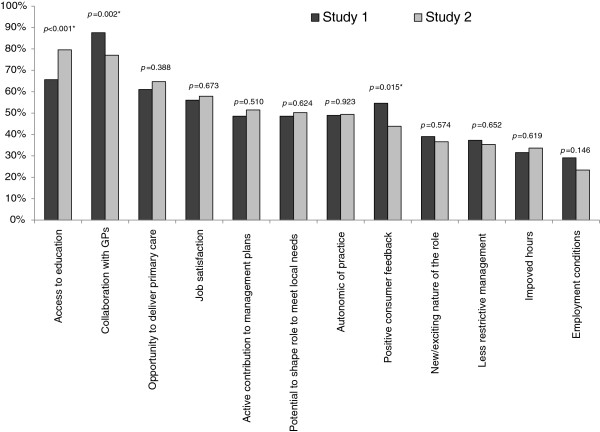
Facilitators’ of practice nurses’ role expansion.

Between the two periods three key changes in the facilitators to role development were apparent. Despite the GPs attitudes being seen as less of a barrier to nurses’ role development, collaboration with the GP was reported less as a facilitator of the nurses’ role in study 2 compared with study 1 (S1 87.6% versus S2 77%; p=0.002). Similarly, positive consumer feedback (S1 54.6% versus S2 43.8%; p=0.015) and employment conditions (S1 29.1% versus S2 23.4%; p=0.146) were reported as a facilitator of role development by fewer participants in study 2 compared with study 1 (Figure 3).

The only facilitator that saw a significant increase between the two datasets was access to education and training. Significantly more participants in study 2 considered access to education and training as a facilitator in their role development compared to study 1 (S1 65.6% versus S2 79.6%; *p<*0.001).

### Levels of optimism

**Figure 4:**
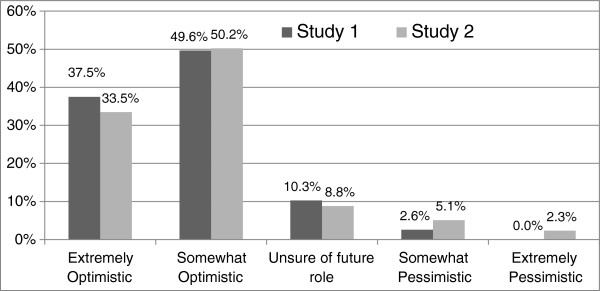
Levels of practice nurse optimism regarding role.

Participants were asked to rate on a five point Likert scale their level of optimism regarding the development of the practice nurse role in Australia. Nurses in both studies continue to have high level of optimism regarding their role expansion (S1 n=237, 87.0%; S2 n=180, 83.7%). The mean score for level of optimism was 4.22 in Study 1 compared to 4.07 in Study 2. However, whilst in Study 1 2.6% (n=7) participants were somewhat pessimistic, in Study 2 5.1% (n=11) participants were somewhat pessimistic and a further 2.3% (n=5) participants extremely pessimistic (Figure 4).

## Discussion

These data provide some salient observations regarding the changing nature of nursing in Australian general practice. Such observations have clear implications for peak bodies and policy makers, as well as for clinicians and consumers. The complexity of the clinical tasks undertaken in general practice is increasing and may be attributable to the increasing numbers of nurses employed and the significant investments in nurse training and development to date [22]. The expansion of advanced and diversified clinical activities is encouraging. However, some key activities continue to demonstrate low confidence amongst nurses within general practice and a need for further education and training. Additionally, in spite of this increase in the complexity of PN role, there was no change in the perception of professional autonomy. The slight increase in pessimism around the role of nurses within general practice and the emerging evidence of poor retention of nurses, may be indicative of a level of frustration around the slow progress in achieving true role development and the continued lack of career pathway for this specialty [22,23]. Despite much attention to such issues in the literature, minimal progress has been made in terms of developing career pathways in the Australian setting. In their Australian study, Parker et al. [23] identified that 85% of practice nurse participants did not have a career pathway in their organisation. Participants also reported a strong feeling that they were regarded as less important than their acute care colleagues [23]. Given the increasing emphasis on providing care within general practice to address the growing burden of chronic disease, there is an urgent need for peak bodies and policy makers to address the workforce issues to promote the retention of skilled, motivated nurses.

It was not clear from this study how many participants had undergone specific formal education programs focussed on general practice nursing. It is recognised that there are limited opportunities for specific formal higher education programs for nurses within general practice [24]. The continuing professional development needs of Australian nurses working in general practice are currently more likely to be met by short courses and workshops [22,24]. Data from this study highlighted the need for focussed education and training to support the nurses’ role.

The limited uptake in postgraduate programs is an impediment to role development as ad hoc training sessions do not provide the structured learning and professional development necessary for advanced roles. In their review of the impact of Masters level education on patient care, Cotterill-Walker [25] identified that graduates demonstrate increased confidence and self-esteem; enhanced communication; personal and professional growth; knowledge and application of theory to practise; and analytical thinking and decision making following completion of their programs. Similarly, Drennan [26] identified that Masters graduates demonstrated significant gains in leadership and management skills as a result of completing their higher degree. Despite the existence of some postgraduate programs for practice nurses in Australia, the uptake of these programs has been variable. Barriers such as cost, lack of familiarity with university education, time commitment and lack of perceived value by practice management have been identified as impediments to PNs undertaking postgraduate programs [22]. As part of developing a career framework, attention needs to be paid to developing a formal education pathway to enhance the clinical and professional skills of nurses in the general practice setting.

Patients’ perceptions of the role of nurses within general practice were seen by the nurses to be much less of a barrier in the second survey. This is supported by findings from both Australia and New Zealand which identify that consumers are largely satisfied with practice nurse services and comfortable with the nurse role in general practice [27-29]. A barrier across both surveys was the perceived lack of collaboration with GPs. This is significant in general practice given the frequent employee/employer relationship between PNs and GPs. It highlights the need for strategies to be implemented to promote the kind of multidisciplinary teamwork that has been demonstrated to improve health outcomes. The concerns expressed by participants in this study about GPs understanding the nurses’ scope of practice, unwillingness to delegate tasks, variation in practice between GPs and unwillingness to delegate are echoed by the experience of McCarthy et al. [24] in Ireland. McCarthy et al. [24] demonstrated that despite some congruence of opinion between GPs and PNs, there remained a degree of divergent opinion regarding the nursing roles, with GPs underestimating the PN scope of practice. Similarly, in their study of culturally and linguistically diverse solo Australian GPs, Halcomb et al. [30] reported that GPs did not feel confident about the roles of nurses and their scope of practice. A factor complicating this issue in the Australian setting is the employment of both EN and RNs in general practice. The different educational preparation and subsequent scopes of practice of EN and RNs adds complexity, particularly for GPs in understanding the nursing role. Whilst there have been attempts to improve teamwork between GPs and nurses, implementing a truly multidisciplinary model of care in Australian general practice requires organisational changes [24,31]. The growing burden of chronic and complex disease facing primary care, and the high level evidence to support the efficacy of multidisciplinary models of care, underscores the importance of actively striving towards such models of care.

This study has a number of limitations. Firstly, the sampling frame and method of this survey challenge the representativeness of this sample. With no means of identifying the population of nurses employed in general practice in either time period it is not possible to calculate a response denominator. Additionally, the sample size is small considering the growing nursing workforce in Australian general practice. However, the sampling techniques used in this study are similar to those used in other Australian investigations and the sample size comparable to others reported in the literature [18,24]. Like any survey, the data collected in these investigations was self-reported and therefore may be subject to recall bias. However, these data provide an important snapshot of trends over time in the Australian general practice nursing workforce. They also underscore the need for data collection methods to monitor issues in human resources for health not just in the general practice setting but globally [32].

Australian general practice is in a dynamic state of growth and faces both challenges and opportunities. As changes occur within this environment these have flow on effects to both the nursing workforce and its role in providing clinical care. There is an increased strategic emphasis on the importance of primary care and primary health care organisations. These data suggest the importance of workforce factors in driving general practice reforms. As in many areas of nursing, retention is a critical concern and this is linked to satisfaction in the workplace. Increasing the emphasis on the specialisation of nursing in primary care will continue to be an important strategic initiative. In order to achieve this, an increased professional profile, including in undergraduate and post graduate nursing courses will be critical. Promoting models of interdisciplinary practice and role definition and refinement may also be of use.

## Conclusion

This study has provided a snapshot across two critical time periods in Australian general practice and provides useful information for nursing workforce planning and models of care. It has identified some of the structural organisational barriers to the nurses role in general practice. The results demonstrate that although strategies to develop workforce capacity have made some inroads to supporting the general practice nurse workforce to grow their role, further attention to workforce development is required. There is a clear need to build structured career pathways with embedded formal practice nurse education programs to facilitate transition of the practice nurse from novice to clinical expert.

These data also emphasise the importance of promoting teamwork and collaborative practice in Australian primary care. They highlight the need to promote interprofessional collaboration and teamwork between GPs and nurses, as well as open discussions between clinicians about how they can best contribute to health care within their professional scope of practice.

## Competing interests

The authors declare that they have no competing interests.

## Author contributions

Study Design (EH, YS, PD), Data Collection and Analyses (EH, YS, PD, RK, SY), Manuscript Preparation (EH, YS, PD, RK, SY). All authors read and approved the final manuscript.

## Pre-publication history

The pre-publication history for this paper can be accessed here:

http://www.biomedcentral.com/1471-2296/15/52/prepub
